# CROSS-NATIONAL DIFFERENCES IN ATTITUDES TOWARD FAMILY AND GOVERNMENTAL SUPPORT FOR ELDER AND CHILD CARE

**DOI:** 10.1093/geroni/igac059.223

**Published:** 2022-12-20

**Authors:** Elinore Avni

**Affiliations:** Boston University, Boston, Massachusetts, United States

## Abstract

Population aging in wealthy western nations has raised concerns about who will provide care to older adults. At the same time, the rise of single parenthood and dual-career families has heightened the need for childcare. As governments and families face challenges in meeting these dual needs, this study compares responses to the question of “who should primarily provide” eldercare and childcare across three countries: the US, Germany and Israel. Analysis of 2012 International Social Survey Programme data reveals that while persons in the US endorse family as care providers to both older adults and children, Israelis endorse government as eldercare providers yet family as the source of childcare provision. German respondents prefer both government and family as childcare providers, yet believe the government should provide eldercare. The paper discusses how cross-national differences in attitudes toward care are associated with cultural and socio-economic characteristics, and highlights implications for policy and practice.

